# Navigating the transition to digital health professions education: a qualitative study of faculty development through the lens of Schlossberg’s transition theory

**DOI:** 10.1186/s12909-026-10761-2

**Published:** 2026-11-13

**Authors:** Miriam Alexander, Martin Lehmann, Harm Peters, Marwa Schumann

**Affiliations:** 1 Dieter Scheffner Center for Medical Education and Educational Research, Dean´s Office of Study Affairs, Charité – University Medical Center Berlin, Charitéplatz 1, Berlin, Germany; 2 Medical Education Department, Faculty of Medicine, Alexandria University, Alexandria, Egypt

**Keywords:** Transition, Faculty development, Digital education, Health professions educators, Qualitative study

## Abstract

**Background:**

Health professions educators must often manage multiple demanding roles as clinicians, researchers, and teachers, while adapting to the transition to digital education. Although faculty development initiatives are designed to support educators in adapting to this changing landscape, there remains a limited understanding of how to navigate this complex transition. Accordingly, this study aims to explore how health professions educators navigate the process of redefining their roles within digital learning environments through their participation in a one-year certified digital faculty development programme.

**Methods:**

This qualitative study utilised focus group discussions and semi-structured interviews with participants enrolled in the Digital Health Professions Educator (DHPE) programme—a 200-hour, certified course designed for experienced educators from diverse health care disciplines at Charité–Universitätsmedizin Berlin. The data were transcribed and analysed using MAXQDA qualitative analysis software. A combined deductive–inductive, content analytic approach was applied, guided by Schlossberg’s (1981) transition theory and its four dimensions: situation, self, support, and strategies. Additional subthemes were identified inductively.

**Results:**

Two focus groups and seven semi-structured interviews were conducted with 15 experienced health professions educators (71% response rate). In the situation dimension, participants described changing roles, teaching motivation, and personal timing, whereas the self dimension highlighted prior online teaching experience and psychological resources as key factors in the transition. The support dimension focuses on peer networks, institutional backing, and mentorship, while the strategy dimension emphasised the importance of professional recognition and the development and application of digital teaching skills.

**Discussion:**

The multifaceted transition towards becoming a digital health professions educator is influenced by the interplay of personal and professional characteristics, the level of digital integration within educational environments, institutional and peer support, and the strategies educators employ to acquire and apply new skills. This shift extends beyond developing technical competencies; it entails a transformation of professional identity, the adoption of innovative teaching strategies, and sustained organisational support.

**Conclusions:**

To effectively facilitate digital transition, faculty development programmes should take a holistic approach that addresses these interconnected dimensions. Schlossberg’s transition theory provides a valuable framework for better understanding the multifaceted nature of this transition.

**Supplementary Information:**

The online version contains supplementary material available at https://doi.org/10.1186/s12909-026-10761-2.

## Background

The transition to digital education is currently reshaping health professions education (HPE) by driving a shift towards the digitalisation of practices and learning environments that complement traditional clinical and pedagogical approaches [1, 2]. This shift presents substantial challenges, as faculty must balance the multifaceted demands of their existing roles as clinicians, researchers, and teachers, while acquiring new digital competencies, pedagogical approaches, and engagements within emerging communities of practice. The transition to the role of digital health educators requires problem-solving, adaptive thinking, and ongoing professional development, all while maintaining standards in their existing areas of expertise [3]. To facilitate this transition, faculty development programmes aim to provide targeted content, structure, and guidance. However, little is known about how health professions educators experience and navigate this multifaceted shift, or how they perceive the effect of such programmes on their own professional practice [4, 5]. This gap in understanding creates the need to explore how educators experience and interpret their transition towards becoming digitally competent professionals. Accordingly, this study explores the experiences of health professions educators as they transition into their evolving roles as digital health educators by participating in a longitudinal faculty development programme.

The global transition to digital higher education, accelerated by the COVID-19 pandemic, has further amplified the role of digital education in the health professions [6–9]. In the field of digital HPE, concepts, pedagogical approaches, and theories are constantly being developed and adapted in response to a multitude of innovations in methods, tools, and implementation frameworks [5, 8]. Several studies have defined core digital competencies for HPE, which include: information management; collaboration, communication, and sharing; creation of content and knowledge; ethics and responsibility; evaluation and problem solving; and technical operations [10, 11]. Digital education has been determined to offer numerous advantages, such as improving student self-regulation and self-efficacy [12, 13], widening the university community [14], and promoting interactive and personalised learning experiences [15]. Notably, the findings reveal learning outcomes comparable to those of face-to-face teaching [16, 17].

Despite the necessity and benefits of digital education, many health professions educators are still navigating their transition towards digital competence and pedagogical confidence [7, 18, 19]. The literature indicates that health professionals predominantly value their roles as physicians and scientists rather than their role as educators, since the latter requires an ongoing career development process involving continual role changes, which can be accompanied by unexpected challenges and obstacles [20–23]. This imbalance is reinforced by institutional support, social recognition, and material rewards typically directed towards clinical and research achievements that inherently foster greater commitment, career focus, and personal investment in these two domains [24]. Consequently, a transition towards more digitally oriented educational practices leaves many health professionals alone facing complex teaching demands beyond any preparation offered by their clinically focused training [25, 26].

To support educators in their ongoing transition towards digitally competent teaching, faculty development approaches emphasise digital teaching skills, digital literacy, and the integration of technology-enhanced learning into programmes [27]. Effective initiatives use various approaches, such as customised e-tutorials, peer-assisted learning, and virtual reality, to develop the digital competence and confidence of educators while addressing barriers such as technical challenges and digital inequalities [8]. Collaborative environments, such as online communities and workshops, facilitate knowledge sharing and reflection, further enhancing pedagogical content knowledge and digital readiness [28]. However, despite these promising strategies, the implementation of effective faculty development programmes often faces challenges owing to a lack of time and incentives, insufficient professional orientation, and organisational and institutional barriers [26, 29, 30].

Digital transformation also requires adequate technological infrastructure, continuous faculty development, and organisational cultures that support innovation, while offering opportunities to enhance accessibility, personalise learning, and prepare health professionals to use digital tools effectively in education and clinical practice. Although considerable attention has been devoted to identifying digital competencies and evaluating faculty development interventions, little is known about how health professions educators understand and navigate the process of professional change associated with digital transformation. Examining this process requires a perspective that extends beyond skill acquisition and captures the dynamic interplay among individual experiences, professional identities and contextual influences. To better understand such transitions, several social science theories and frameworks have been proposed to explain how to navigate change and respond to the evolving professional demands. In Bridges’ Transition Model, transition is conceptualised as a psychological process that unfolds through phases of letting go of previous ways of working, navigating a period of uncertainty, and eventually embracing new roles and practices [31]. In contrast, Meleis’s Transition Theory focuses on the experiences and conditions that influence individuals’ responses to change [32].

Complementing Meleis’s perspective, Schlossberg’s Transition Theory (STT) provides a structured lens to examine the interactions among personal characteristics, situational circumstances, available support systems, and coping strategies [33]. According to STT, an individual’s ability to navigate and adapt to periods of change is influenced by four interrelated dimensions. The situation dimension encompasses the characteristics and circumstances surrounding the transition, including its timing, context, and perceived impact; the self dimension refers to the personal and psychological attributes that shape an individual’s interpretation of and response to change; the support dimension considers the availability and accessibility of interpersonal, organisational, and social resources that can facilitate adaptation; and the strategies dimension comprises the cognitive and behavioural approaches individuals use to manage challenges, cope with uncertainty, and handle the demands associated with transition. Applied to digital HPE, these dimensions enable consideration of the circumstances surrounding digital transformation, educators’ personal resources and prior experiences, the institutional and collegial support available to them, and the strategies they use to adapt their teaching practices.

Several studies have used STT to explore the transitions of health care professionals, such as the transition of physicians becoming medical educators [20]; the transition of first-year medical, dental, and pharmacy students at university [34]; and the transition of faculty from current to future educator roles [35].

For the present study, STT was selected because its multidimensional focus provides a structured lens for examining how educators navigate professional change through the interplay of contextual circumstances, personal resources, available support, and adaptive strategies, making it particularly relevant to the transition to digital teaching and learning. The study aims to explore how participation in a structured, longitudinal faculty development programme supports educators in navigating this transition and to generate insights that may inform the development of faculty development programmes supporting digital change. This study is guided by the following research question: How do health professions educators experience the transition into digital teaching roles through participation in an intensive faculty development programme?

## Methods

### Philosophical assumptions

This qualitative study is informed by social constructivist epistemology theory, which posits that knowledge is co-constructed through human interaction and shaped by social and cultural contexts. This theory was employed to explore how health professions educators navigate the transitions related to digital teaching and the evolving professional roles within the dynamic landscape of HPE, as experienced through social interaction [36]. In line with this view, we approached the generation of knowledge as a collaborative process among ​participants wherein meaning is constructed through dialogue, reflection, and interpretation [37].

### Study setting and participants

**Fig. 1:**
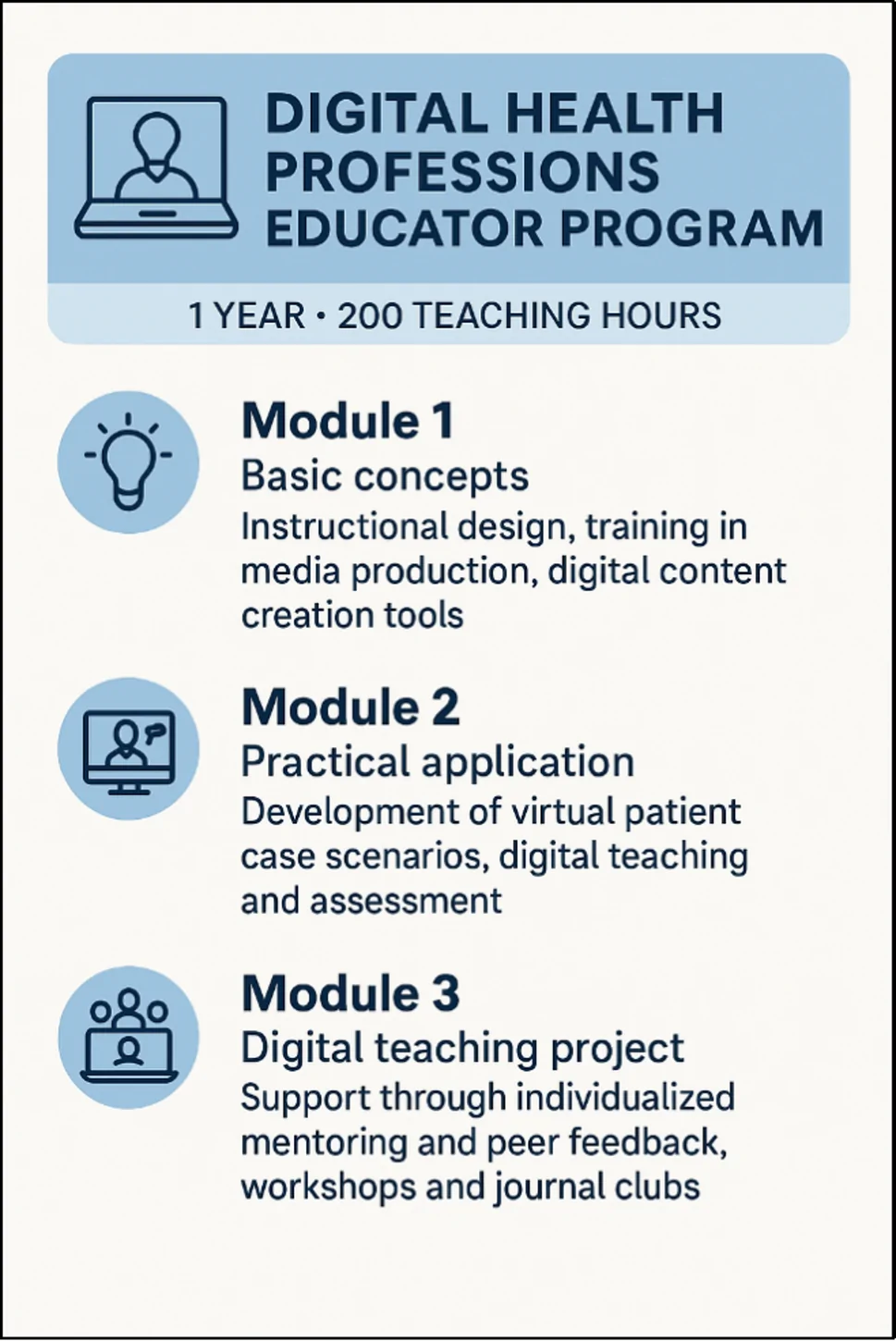
Overview of the Digital Health Professions Educator programme

The study was conducted at Charité - University Medical Center Berlin​, a large medical facility in Europe. As part of the Digital Health Professions Educator (DHPE) programme, this study explores the lived experiences of health professions educators as they navigate a complex transition into expanding roles in digital education, highlighting the interplay among personal, institutional, and strategic factors in shaping their professional development. The DHPE programme aims to expand individual qualifications with the acquisition of a final certificate, and to enable participants to act as multipliers to promote faculty development in the field of digital teaching. The programme is a year-long commitment, encompassing 200 teaching hours that are divided into three modules (Fig. 1). The DHPE is intended for lecturers from all Charité study programmes (including human medicine, dentistry, health science, nursing, applied midwifery and several postgraduate courses such as public health and medical neuroscience). In addition, participation in the DHPE requires proven pedagogical experience, the submission of an application, and a letter of motivation. Educators who participated in the (DHPE) programme were recruited for this study using a purposive maximum variation sampling strategy to maximise the range of experience [38, 39]. Participant recruitment targeted health professions educators enrolled in the DHPE programme who met the programme’s admission criteria, including documented teaching experience, submission of an application, and a motivation letter. All eligible participants from the two DHPE cohorts (2023/2024 and 2024/2025) were invited to participate in the study through recruitment emails and personal invitations during face-to-face programme sessions. Participation was voluntary, and no financial compensation was provided.

### Qualitative data collection, transcription and translation

This qualitative study included virtual focus group discussions and semi-structured interviews (MS Teams Vers. 24193.1707.3028.4282, © Microsoft 2024) with an interviewer not involved in the conception or implementation of the DHPE programme (M.A.) in the summer of 2025. We interviewed all educators who replied and consented. The interview questions explored the participants’ motivation to participate in the DHPE programme, their learning experiences, their growth in personal competencies and skills, their transfer into teaching practices, their programme satisfaction, and their suggestions for improvement (see Supplemental Appendix 1 for the interview guide). Prior to data collection, the interview guide was piloted and refined to improve the clarity and relevance of the questions. All interviews and focus group discussions were audio-recorded and transcribed verbatim by the author (M.A.) using MS Word (Word 2021, Microsoft 365). The transcripts were reviewed and corrected by a research assistant and the interviewer. For structured and comprehensible transcription, the adapted transcription rules of Kuckartz (2014) were applied. The interviews and focus groups were conducted in the German language. The listed quotes for the purposes of this article were translated into English by the author (M.A.).

### Qualitative content analysis

Data collection, transcription and ana​lysis occurred simultaneously in an iterative process [40, 41]. Content analysis was chosen as the methodological approach because of its suitability for capturing participants’ perspectives and facilitating thorough, systematic data interpretation. Consistent with the social constructivist epistemology underpinning this study, meanings were understood as being interpretively developed through participants’ accounts and the researchers’ analytical engagement with the data, allowing for a nuanced understanding of the transition experience. The analysis followed a combined deductive–inductive approach. At the deductive level, Schlossberg’s Four-S Framework was used to define the main coding categories a priori: situation, self, support, and strategies.

These categories provided the initial structure for organising and interpreting the data in relation to the study’s theoretical framework. Within each main category, subthemes were then developed inductively from participants’ accounts, allowing the analysis to remain open to patterns and meanings that emerged from the data [39, 42]. This combined deductive–inductive approach enabled us to apply Schlossberg’s 4 S Framework to the context of digital HPE while remaining open to context-specific subthemes emerging from participants’ accounts [33, 43].

We followed the key stages of qualitative content analysis, beginning with de​contextualisation, in which the first author (M.A.) segmented the transcripts into meaningful units and assigned codes to significant phrases or concepts that aligned with the research question. Rather than relying solely on the concept of data saturation, which has been critiqued for reflecting more positivist assumptions within qualitative research, sampling adequacy was guided by the principles of information power, conceptual depth, and theoretical sufficiency [44]. Data adequacy was considered iteratively throughout data collection and analysis, as were the focused study aim, the use of an established theoretical framework, and the inclusion of participants who participated in the DHPE programme. The coding was initially based on two interviews and two focus group transcripts, after which no substantially new relevant insights were identified, indicating information redundancy and sufficient information power for the analysis [45]. Additional interviews were conducted to include perspectives from both DHPE cohorts and to further enrich the variation and contextual depth of participant accounts. This was followed by recontextualisation, where a review of the codes was undertaken by the research team (M.S. and M.L.) to ensure that they accurately reflected the data. In the categorisation phase, the research team (M.A., M.S. and M.L.) grouped the data according to the initial deductive coding structure derived from Schlossberg’s 4 S Framework, using the a priori themes of the original framework as main themes while inductively identifying emerging themes as subthemes within these categories based on participants’ accounts. In the final compilation phase, there was a considerable amount of overlap between categories, which was discussed by the research team (M.A., M.S., M.L. and H.P.) on the delimitation of individual categories and the significance of overlaps with respect to the interpretation of the results. The author (M.A.) reviewed all coded transcripts. The allocation of quotations to the definitive categorisation system was undertaken by the author (M.A.) to facilitate the discussion of results within the team (M.S., M.A. & M.L.), with the discourse being informed by general impressions, noticeable patterns and interpretations. At all stages of analysis, the authors collaboratively reviewed the coding and interpretation of the data through regular analytic meetings. To enhance the trustworthiness and consistency of the analysis, coded excerpts and emerging themes were discussed iteratively among the research team, with particular attention given to areas of ambiguity or differing interpretations. Disagreements were resolved through reflexive dialogue, comparisons with the original transcripts, and negotiation until consensus was reached among all authors, as is consistent with established approaches to collaborative, qualitative analysis and researcher triangulation [46]. For coding and data analysis, we employed MAXQDA software (MAXQDA 2018.1). The steps from study planning and data collection to data analysis in iterative team discussions were documented in the study protocols.

### Reflexivity, quality and rigor

Reflecting critically on the relationships between researchers and participants is fundamental to upholding the rigour and integrity of a research process [39, 40]. As an interdisciplinary research team comprised of researchers of different genders and characterised by diverse professional, national, linguistic, and cultural backgrounds, as well as varying levels of expertise in qualitative research, we engaged in critical reflexivity regarding how these characteristics and experiences may have influenced the scope, interpretation, and depth of the study.

Both authors (H.P.), a senior professor with extensive experience in medical education and research project management, and (M.S.), a qualitative medical education research specialist, hold medical degrees and Master’s qualifications in medical education. The author (M.L.) has a background in nursing and HPE, and the author (M.A​.) is a psychologist whose view of the data is determined by the organisational psychologist’s view of change processes.

Three members of the team (H.P., M.S. and M.L.) can be considered ‘insiders’: the author (H.P.) was directly involved in the programme’s conceptualisation and development, while the authors (M.L. and M.S.) contributed to its implementation through teaching activities. To balance the authors` proximity to the programme and mitigate potential bias, the author, therefore not involved in the validation of transcripts or findings.(M.A.) had no prior involvement in the programme and no direct relationship with any of the study participants. As an ‘outsider’ (M.A.) was able to contribute a degree of analytical distance and objectivity to the research process. To further enhance reflexivity and transparency, the team maintained a research diary throughout the study to document reflections, methodological decisions and evolving interpretations. In addition, the interdisciplinary research team held frequent meetings to encourage robust discussions and the integration of diverse perspectives, thereby promoting a more comprehensive and balanced interpretation of the data.

Credibility, which corresponds to internal validity in qualitative research, was supported through triangulation by involving multiple researchers in the data analysis (researcher triangulation) [46]. Additionally, data were collected from two different programme cohorts and included participants with diverse professional backgrounds, thereby enhancing the breadth and depth of the findings (data triangulation) [47]. Member checking was not undertaken, as participants had completed the DHPE programme and graduated prior to the analysis phase. Hence, they were therefore not involved in the validation of transcripts or findings. To enhance credibility, we instead relied on iterative data collection and analysis, regular discussions among the interdisciplinary research team, and the use of verbatim quotations to support the interpretation​ of the findings. Furthermore, the research questions, the underlying epistemological framework, and the chosen methods were aligned to reinforce methodological rigour and promote conceptual coherence and validity [48].

### Ethical considerations

This study was conducted in accordance with the ethical principles of the Declaration of Helsinki. Ethical approval was obtained from the data protection and ethics committee of Charité - University Medical Center Berlin (r​eference no. 402505). All the participants were informed of the purpose of the study, the voluntary nature of their participation, and their right to withdraw at any time without adverse consequences. Written informed consent was obtained from all participants prior to data collection. It was explicitly communicated that participation in the study would not affect their success or progression within the programme. To ensure confidentiality and anonymity, all transcripts were pseudonymised, and identifying information was removed during the transcription process. Data were securely stored in password-protected files, accessible only to the research team. Participants were also informed that their anonymised data may be used for publication and further research related to faculty development and digital education in health professions.

## Results

### Demographic data of the study participants

A total of 15 participants (seven women and eight men) participated in two focus groups (58–64 min) and seven semistructured interviews (29–55 min). Seven of the participants were from the 2023/2024 cohort, and eight were from the 2024/2025 cohort, yielding a response rate of 71% from the 21 invited participants. The participants represented a broad range of career stages and an age composition ranging from 30 to 55 years, including early-career educators and postdoctoral researchers (*n* = 4), mid-career clinician educators and teaching coordinators (*n* = 8), and senior faculty members with longstanding experience in academic teaching and educational leadership (*n* = 3). The participants also represented a wide range of disciplines, including 2 each from anaesthesiology and emergency medicine, and one each from paediatrics, geriatrics, infectious disease, clinical nursing, nephrology, pharmacology, IT/scientific computing, pathology, medical physics, biology, and social medicine.

### Coding Framework

**Fig. 2:**
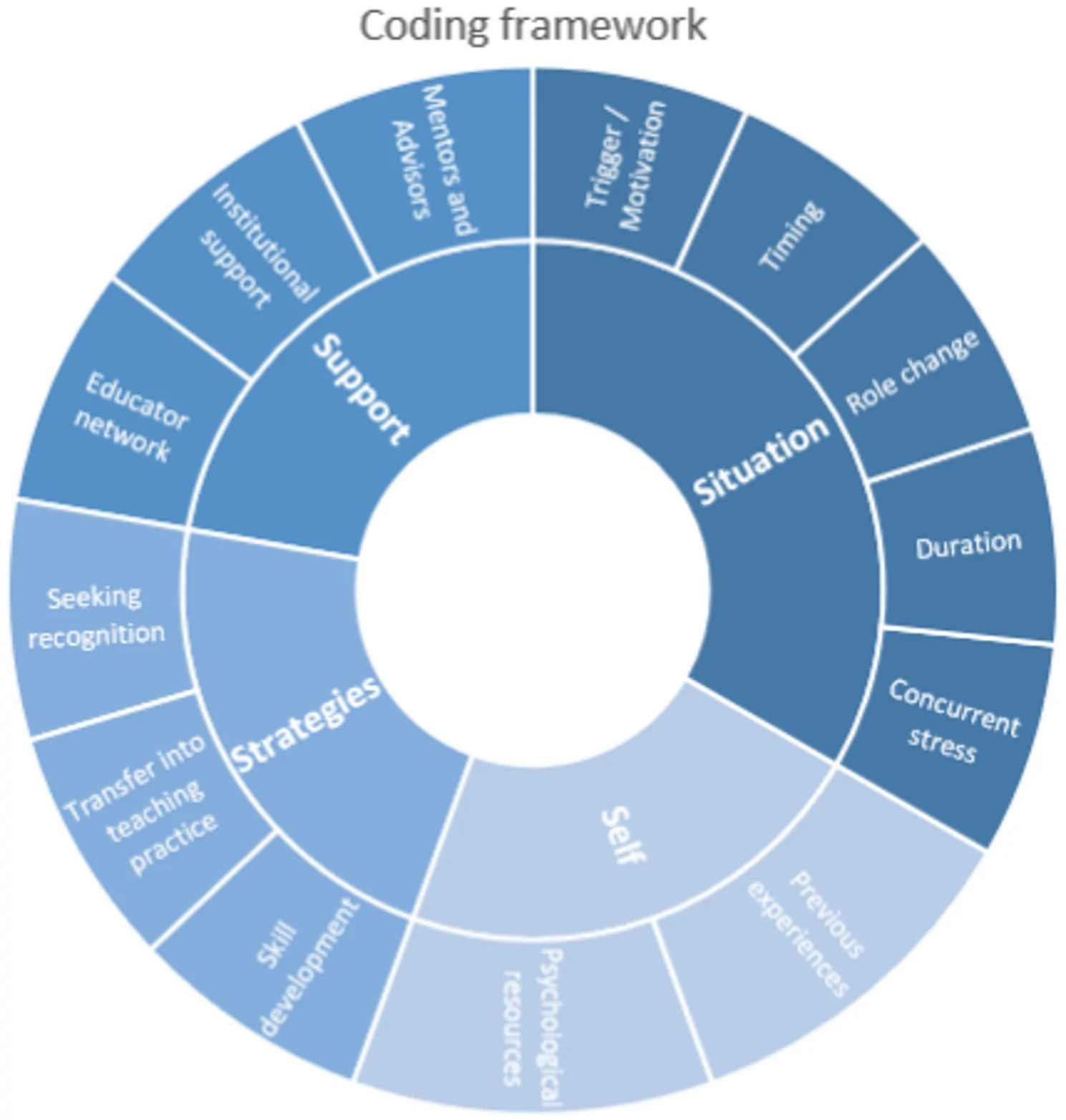
Coding framework adapted from Schlossberg’s Four S (4 S) Model

The coding framework was composed of Schlossberg’s four central transition dimensions as main themes: situation, self, strategies, and support (see Fig. 2). Subthemes within the situation category, including trigger/motivation, timing, role change, duration, and concurrent stress, as well as subthemes within the self category, including previous teaching experience​ and psychological resources, aligned closely with the original dimensions of Schlossberg’s framework. Additional emerging subthemes were identified inductively, particularly within the strategies category, including digital skill development, transfer to teaching practice, and seeking of recognition, and within the support category, including educator networks and institutional support for digital teaching. These emerging subthemes reflected the contextual aspects of digital HPE and faculty development that were less explicit in the original framework. Participants’ accounts indicated that the themes were not experienced in isolation. Rather, they revealed relationships among the four dimensions, i.e., situation, self, support, and strategies, with experiences in one area often influencing or shaping experiences in another.

### Theme 1: Situation

The situation theme explores the contextual conditions under which participants engaged in the transition towards digital HPE. Rather than representing a single event, participants described the transition as an ongoing process shaped by the interactions among personal aspirations, career circumstances, evolving professional expectations, and workplace constraints. Five interrelated subthemes were identified: trigger/motivation, timing, role change, duration, and concurrent stress.

#### Trigger/Motivation

Participants engaged in digital health profession education to modernise their teaching practices and respond to evolving educational demands. Their motivation extended beyond acquiring technical skills, reflecting a desire to improve teaching quality, innovate pedagogical approaches, and address perceived gaps in their expertise. They frequently initiated the transition through critical reflection on existing teaching practices, recognising that traditional approaches were no longer sufficient for contemporary learning environments.

“My primary motivation was to improve and further develop my competencies regarding the use of digital media and teaching. The requirements for instructors using digital or blended programmes differ from those associated with traditional methods” #14.

#### Timing

Participants’ broader professional trajectories strongly influenced their engagement with faculty development, which was shaped by career transitions and long-term career planning. These findings suggest that readiness for transition is closely linked to educators’ perceptions of how digital teaching competencies align with their future professional goals, and the development of their professional identity.

“And, as I said, I personally want to see how I can further develop my career, and I would very much like to move more into teaching and continue building on that. I now see this as a good opportunity to move in that direction.” #8.

#### Role change

A central feature of the transition was the reconsideration of participants’ professional roles, where becoming a digital health professions educator was described as expanding​ and legitimising the educator role alongside established identities as clinicians and researchers, and this served as a catalyst for strengthening their professional identity as educators and redefining their contribution to the academic community.

“Well, we are physicians, but we have many other roles in our daily work. The role of teacher, however, was never truly taught to us, so we, more or less, just carry it out on our own.” #5.

“When you look at it and ask: ‘What does it actually mean when I say I am a digital health professions educator?’ — that is indeed an ambitious goal.” #2.

Participants framed this expansion of roles not only as a personal aspiration but also as a collective responsibility, emphasising the importance of “passing on” knowledge to colleagues and contributing to wider professional networks:

“Of course, we are involved in several degree programmes […] and now we have a group for ourselves, and perhaps one of our tasks is to pass on this information.” #5.

#### Duration

Participants viewed the transition as an ongoing developmental process rather than a final learning outcome. Although the DHPE programme supported the development of competencies, educators recognised that the rapidly evolving nature of digital technologies requires continuous adaptation. Discussions about artificial intelligence revealed that participants viewed digital competence as a moving target, requiring sustained engagement rather than mastery of a fixed set of skills. This highlights the dynamic nature of the transition, where educators must continuously reassess their competencies in response to technological developments and changing educational contexts.

“[…] when you think about what I would formulate as a course objective if I take 2024. Then, again, I might have some criticism. What about AI, for example? […] That did not come up, and the course would, of course, have to determine whether that [AI] actually belongs in DHPE or not.” #2.

“I believe that within the broad field of digitalization, there are, of course, still areas where one does not feel fully competent or thinks things could be done better or faster. However, I feel that the course has motivated me to keep working on it.” #11.

#### Concurrent stress

Because the transitions occurred in professional environments that were characterised by competing demands and limited resources, participants’ ability to engage in educational development was frequently constrained by clinical responsibilities, administrative duties, and unpredictable workloads. These pressures were intensified by a perceived lack of institutional recognition for teaching activities. Therefore, the findings suggest that situational barriers were not merely practical challenges; rather, they actually reflected broader tensions between educational aspirations and organisational priorities.

“Speaking from the clinical perspective, one obstacle is the sudden need to cover shifts. This may involve taking on teaching duties because someone is ​absent. Such unforeseen substitutions, especially in daily clinical work, or simply not having time on a given day because one has to step in elsewhere, are, I believe, the greatest challenges for me and probably for several others as well.” #9.

Taken together, the Situation theme shows that the transition to digital HPE was influenced by the interplay of personal aspirations and contextual constraints. That is, intrinsic motivation and career timing created opportunities for engagement, while competing professional demands influenced how participants experienced and sustained the transition. These findings suggest that becoming a digital health professions educator involves not only individual learning, but also the negotiating of evolving professional roles within complex organisational environments.

### Theme 2: Self

The self dimension explores how participants’ prior experiences and psychological resources shaped their engagement with the transition to digital HPE. The analysis revealed that educators’ responses to the transition were influenced by their existing teaching experiences, as well as by their ability to tolerate uncertainty, stay motivated, and maintain effort over time. Two interrelated subthemes were identified: previous teaching experiences and psychological resources.

#### Previous teaching experiences

The participants perceived a discrepancy between their expertise as health professionals and their preparedness for educational roles. Although they were confident in their disciplinary knowledge, many described entering teaching with little formal preparation in pedagogy, curriculum design, or learner-centred approaches. This lack of educational training created a sense of professional uncertainty, prompting participants to reflect on their teaching practices and seek development opportunities.

“Another issue was the absence of formal preparation in effective teaching methods. We feel confident in our subject knowledge; we are not adequately trained in how to convey this knowledge to students. […] The implicit assumption appears to be that proximity to one’s own studies is sufficient to teach. This is frustrating. […]” #4.

Rather than acting as a barrier, these experiences often served as a catalyst for engagement with faculty development. Participants described how the rapid shift to online teaching during the pandemic exposed the limitations of their existing approaches, highlighting the need for more deliberate pedagogical and digital competencies. Thus, the transition appeared to be driven not only by technological change, but also by a growing awareness of teaching’s complexity as a professional practice. These findings suggest that prior teaching experience functioned as an important interpretive lens through which participants evaluated their own readiness for digital education:

“We delivered teaching well before the pandemic, and we continued teaching during the pandemic—whether that was good or bad, we still cannot truly say. However, these experiences make it clear that it would be valuable to ‘fill the skills toolbox.’” #2.

#### Psycholo​gical resources

Participants described the transition as unfolding in an environment of uncertainty and continual change. Instead of expecting stability, they acknowledged that unpredictability is an inherent feature of digital innovation and academic work. This finding suggests that successfully engaging with the transition depended not only on acquiring new competencies, but also on developing psychological capacities that enable adaptation in uncertain situations.

“These are exactly the unforeseeable disruptions, and because they cannot be planned for, one simply has to see what to do in the given situation. Personally, I do not expect anything predictable, so to speak. However, nothing can be ruled out.” #8.

Notably, participants did not frame these qualities only as individual personality traits, but rather as professional resources that enabled them to navigate the ongoing demands of digital transformation. Their accounts suggest that passion and sustained motivation played central roles in overcoming the challenges associated with expanded roles, increased workloads, and changing teaching expectations:

“Well, as my supervisor always puts it nicely, it is a question of passion. The time required is simply invested. I would describe myself as someone with endurance; if something needs to be done, it will be done.” #2.

In summary, the Self category illustrates how participants’ transition experiences were shaped by the interaction between their prior educational experiences and their internal psychological resources. Although deficiencies in pedagogical preparation promoted professional development, qualities such as adaptability, perseverance, and commitment allowed participants to persist through the transition, despite uncertainty. These findings suggest that educators’ responses to digital transformation are influenced by external circumstances and by how they interpret and utilise their personal resources in response to change.

### Theme 3: Strategies

The Strategies theme e​xplores how participants responded to the demands of transitioning to digital HPE. Rather than describing skill acquisition as an end in itself, participants framed the use of strategies as an ongoing process of experimentation, adaptation, and professional positioning. The analysis reveals that participants navigated the transition by developing new competencies, integrating them into their work through iterative refinement, and seeking recognition for their educational efforts. Three interrelated subthemes were identified: skill development, transfer into teaching practice, and seeking recognition.

#### Skill development

Participants viewed the development of digital competencies not simply as learning new technical tools, but as broadening the range of teaching methods and educational approaches available to them. They reported that learning to use digital platforms, content creation tools, and interactive applications increased their perceived ability to design and facilitate digital learning environments.

“Well, I think I was truly able to improve in the area of video and image editing. I had already been interested in it before, but I just hadn’t truly had the opportunity to engage with it more in depth.” #11.

“Well, my main goal​ was definitely to develop a good command of online tools. I would also have liked to be introduced to different video conferencing platforms.” #2.

#### Transfer into teaching practice

Participants described applying their newly acquired digital competencies as an ongoing process that went beyond learning to use specific tools. They reported that the real challe​nge is in adapting these competencies to their own teaching contexts, and making practical decisions about how to implement digital formats. For instance, participants discussed the challenges of creating concise video content for blended learning, and of balancing content coverage with format requirements.

“Well, we truly struggled with how to manage creating a short presentation video, approximately 8 minutes or so, in order to, for example, implement blended e-learning — not endlessly long, but short, approximately 10 minutes or something like that.” #3.

Participants further described implementation as an iterative process that involved experimentation and adjustment. Rather than applying newly acquired skills directly, they reported learning through the process of using digital tools in their teaching projects and identifying challenges as they arose. This iterative engagement with technological tools enabled participants to adapt their pedagogical approaches in response to both the opportunities and the constraints of digital teaching environments.

“The transfer into the teaching project was, in parts, still rather tedious, but it was really a matter of having to actually do it and try it out — only then do you see, so to speak, where the ‘pitfalls’ lie […]. It simply takes time.” #5.

#### Seeking recogniti​on

The importance of recognition was highlighted by participants, with certification being perceived as formal acknowledgement of their efforts and validation of their commitment to teaching. It was also considered to be essential for their professional identity and acknowledgement within academic communities. That being said, participants emphasised the lack of recognition of teachers’ commitment, which was also evident by the lack of protected time for teaching responsibilities.

“…The issue of appreciation isn’t really the point here. The [DHPE programme] was done in our spare time, and it still hasn’t received much attention.” #12.

This suggests that recognition served both individual and organisational functions. At the individual level, recognition reinforced educators’ commitment to teaching and contributed to​ the development of their professional identities, while at the organisational level, the absence of recognition was perceived as a barrier to sustaining engagement with educational innovation.

In summary, the Strategies theme illustrates how participants navigated their transition by developing digital competencies, gradually integrating new approaches into their teaching practices, and seeking recognition for their educational contributions. These interconnected strategies enabled educators to translate their newly acquired skills into practice through ongoing implementation and refinement. Notably, participants’ engagement with digital tools and platforms was closely linked to their decisions about teaching design and delivery, whereas formal recognition helped sustain their commitment to their evolving role as digital health professions educators.

### Theme 4: Support

The support theme explores how social and organisational resources influence participants’ transition to digital education in health professions. They described drawing on support from peers, institutional structures, and DHPE teachers and mentors. Each source of support contributed differently to their engagement with the programme and their ongoing development as educators. Three subthemes were identified: educator network; institutional support; and mentors, advisors, and DHPE teachers.

#### Educator network

Participants consistently emphasised the importance of peer networking, which was experienced as both a practical and emotional source of support, allowing participants to share ideas, discuss challenges, and feel part of a learning community.

“I found the idea of networking very important; it truly matters to me and is something close to my heart.” #8.

The value of the peer network was particularly evident in participants’ descriptions of shared experiences. Coming together with colleagues helped participants recognise that their worries, needs, and frustrations were not individual problems, but rather, they were shared by others in similar teaching contexts:

“I think we can all agree on that. I believe the communal aspect—coming together, sharing experiences, and realising that one is not alone with their own worries, needs, and frustrations—was a major part of the course and helped all of us, certainly me. […]” #4.

#### Institutional support

Participants described institutional support ambivalently, reporting both enabling and constraining conditions that influenced their engagement with the digital teaching programme. They perceived support from supervisors and dedicated time for participation as important facilitators that created opportunities to engage with the DHPE programme despite competing professional responsibilities.

“Well, in my case, my supervisor said he thought it was a good idea and that I should do it. Once he said that, it was guaranteed that the necessary time frame would be provided.” #7.

Participants also identified the DHPE programme as a valuable institutional resource. In contrast to isolated faculty development activities, the programme was perceived as providing a coherent and longitudinal approach to educational development, which participants considered particularly beneficial for supporting ongoing learning:

“I am very satisfied that this programme exists or existed, because, unlike the singular didactics courses, it provided a consistent and higher education didactic […] let’s say, coherence in teaching. I believe that is something which represents a unique characteristic, if you will — a unique selling point of the DHPE programme […]**”**#14.

However, participants highlighted limitations in institutional support that constrained their ability to sustain engagement with educational innovation. Although they described their motivation to teach as high, they emphasised that motivation alone could not compensate for the lack of time, staff, and workload support:

“Therefore, if you want to have good teachers, you must, on the one hand, give them more time, and on the other hand, also provide them with people who are available to help with the workload, because the ones who are there are highly motivated, but they do have their limits.” #1.

Participants also identified challenges related to institutional coordination, and perceived a lack of awareness of colleagues’ activities as contributing to duplicated efforts and missed opportunities for collaboration:

“Well, I can imagine that some ideas are duplicated, or work is doubled, simply because one is not aware of what others are doing. I think there is still a lot of potential to create synergies there” #5.

The influence of institutional support was closely connected to other dimensions of the transition process. Participants’ ability to apply newly acquired digital competencies (strategies) often depended on the availability of time, resources, and organisational support, whereas institutional constraints frequently intensified the workload pressures described within the situation domain. Similarly, supportive supervisors and structured faculty development opportunities appeared to reinforce participants’ motivation and commitment to continued development.

#### Mentors and advisors/DHPE teachers

Participants also identified DHPE teachers and course leaders as important sources of support during transition. Their roles extended beyond simply delivering content, to include their motivation, availability, and interactions with participants.

“Yes. I definitely had the impression that the people teaching the programme were all very motivated to contribute. […] One could truly sense that there was a very good exchange between the instructors and the learners.” #1.

“To emphasise this now, perhaps […] Well, I found the course leader […] to be quite inspiring. […], because she […] had a good manner somehow and also always contributed something additional, and she also adjusted herself to the participants.” #13.

In summary, the Support theme illustrates that participants’ transition into digital HPE was shaped by peer networks and institutional structures that enabled participation but also revealed gaps in time, staffing, and coordination. Moreover, DHPE teachers provided guidance and relational support throughout the transition process. Across all four domains, personal resources, contextual conditions, available support, and adaptive strategies were determined to be interconnected. Rather than operating as separate dimensions, these factors appeared to interact and collectively shape participants’ experiences as they transitioned to becoming digital health professions educators.

## Discussion

The transition to becoming a digital health professions educator is a multifaceted process that extends beyond the acquisition of technical competencies. Our study concludes that this transformation is shaped by the interplay of professional roles, institutional culture, personal motivation, and strategic adaptation. Viewed through Schlossberg’s transition theory, the findings suggest that the four dimensions do not operate independently; rather, they are closely interconnected. Participants’ accounts indicate that situational challenges often activated personal resources, whereas the availability of support influenced the extent to which new strategies could be developed and applied. In turn, experiences gained through implementing new approaches shaped how participants perceived subsequent challenges and opportunities. This highlights the dynamic and iterative nature of the transition process, in which different dimensions of the transition continuously interacted over time. While Meleis’s Transition Theory provides a broader perspective on transition experiences, conditions, and patterns of response, it is less explicitly structured around the resources individuals mobilise to navigate change. Schlossberg’s framework, in contrast, specifically organises these resources through Situation, Self, Support, and Strategies, although it places less emphasis on transition outcomes. ​ In the context of digital HPE, this focus offered a particularly relevant lens for examining how educators navigate changes in their professional and teaching practices through personal and contextual resources, support, and adaptive strategies. Accordingly, by situating these findings within the broader literature and by using the four S themes of Schlossberg, the implications of those themes in supporting the sustainable digital transition of health care professionals are explored.

With respect to the ​Situation theme, our findings indicate that the engagement of healthcare professionals in the transition to digital HPE was shaped by a combination of intrinsic motivation, timing within their career trajectory, and concurrent workload pressures. These findings align with those of previous studies that emphasise the important role of situational context, including workload and institutional timing, in shaping educators’ readiness and capacity to adopt digital teaching practices [49]. Similarly, research on faculty digital competence has determined that while external triggers, such as the COVID-19 pandemic, accelerated digital transitions, such triggers also heightened stress and time constraints, thus highlighting the dual nature of situational factors as both catalysts and barriers [6, 7, 50–52]. In contrast, other studies have reported that, once educators have developed strong digital skills and institutional structures are firmly in place, situational challenges such as workload and timing have less of an impact [28, 53]. Our findings thereby extend the literature by illustrating the dynamic interaction between educators’ motivational readiness and their professional and institutional context, suggesting that favourable timing and intrinsic motivation can enable sustained engagement despite workload demands.

With respect to the Self theme, our findings reveal that prior experience, adaptive capacity, and psychological resources, such as perseverance and openness to change, played important roles in shaping how health professions educators engaged with the transition to digital teaching. These results align with those of previous studies that have demonstrated the significance of personal qualities, i.e., self-efficacy, openness to change, and reflective capacity, in facilitating educators’ successful engagement with digital teaching innovations [54]. In contrast, other studies have suggested that personal traits alone are insufficient to ensure digital teaching readiness, and instead have argued that structural and institutional factors, such as workload and resource availability, play a more decisive role [30]. Our results advance the understanding of digital transitions by demonstrating that self-related factors play an important role in mediating the relationship between situational conditions and educators’ engagement in the transition process. Educators who demonstrated adaptability, perseverance, and intrinsic motivation reported greater ability to manage workload pressures and uncertainties. In contrast, educators with less experience or confidence reported relying more heavily on external support structures to maintain progress in developing digital teaching practices.

With respect to the ​​Support​ theme, our findings indicate that peer exchange, institutional backing, and mentorship were essential for facilitating the transition to digital HPE. The opportunity to share experiences and ideas with colleagues fostered a community of practice that improved motivation and reduced feelings of isolation. This aligns with previous research that emphasised the importance of collaborative networks and structured institutional support in promoting engagement and sustaining change in digital teaching [41, 55]. Similar to previous studies, our study concludes that institutional support through structured faculty development, adapted training based on self-assessment feedback, and designated support teams enabled health professions educators to overcome challenges, such as limited digital competence, technical hurdles, and engagement issues [48, 49]. Accordingly, our results suggest that effective support extends beyond formal institutional mechanisms and includes social and relational elements, such as collegial exchange and accessible mentorship, which enable educators to translate digital competencies into lasting teaching practices.

With respect to the Strategy theme, our findings reveal that digital skills were developed through hands-on engagement and their gradual application while teaching, with formal recognition being an important motivator. These results are consistent with evidence suggesting that developing skills in digital education is most effective when it is based on active experimentation and reflection within authentic teaching contexts [7]. Research into faculty development consistently demonstrates that opportunities for practice and feedback increase confidence and facilitate the adoption of new teaching methods. Furthermore, our findings on certification as a form of acknowledgement are consistent with the concept that recognition mechanisms, such as credentials, teaching portfolios, and institutional promotion criteria, reinforce engagement and legitimise teaching as a valued academic activity [53]. Conversely, studies that highlight the negative effects of limited institutional incentives and high workload demands emphasise that, without structural reinforcement, motivation and skills risk being short lived [56]. Taken together, these insights suggest that effective faculty development requires more than just skill acquisition. It also necessitates iterative practice, structured application, and visible institutional recognition to foster long-term engagement within the digital teaching environment. More broadly, these findings position digital HPE as an organisational as well as an individual, transition, requiring coordinated attention to curricula, teaching and assessment practices, and faculty development to support the sustainable integration of digital approaches.

Although the primary focus of this study was on educators’ transition processes, our findings also indicate that engagement with specific digital tools and platforms played an important role in shaping teaching practices. For example, participants’ experiences with technologies such as video conferencing tools, content creation software, and emerging applications such as artificial intelligence influenced not only skill development, but also pedagogical decision making. These findings suggest that technological affordances are intertwined with professional transition processes, as educators adapt both their practices and their identities in response to evolving digital environments.

Overall, Schlossberg’s 4 S Model was found to be a very useful psychosocial framework for exploring and analysing the multifaceted transition process of becoming a digital health professions educator. In line with Schlossberg’s concept, our findings demonstrate a close interrelationship among the four dimensions, i.e., situation, self, support, and strategies. More specifically, situational triggers such as career timing and workload, influenced educators’ perceived readiness for change by interacting with personal characteristics, such as resilience and motivation. Support from peers, mentors, and institutions was crucial in facilitating the adoption of strategies for developing and applying digital teaching competencies. While the framework provided a useful deductive structure for analysing how HP educators navigated changing teaching roles, digital learning environments, and evolving professional responsibilities, the inductively derived findings provided additional context-specific insights into digital faculty development, particularly regarding the application of digital teaching practices, the integration of digital competencies into educational work, and the role of institutional and collegial structures in supporting these processes. By applying Schlossberg’s framework to the context of digital faculty development, this study provides a context-specific understanding of how health professions educators navigate professional transitions in digitally evolving educational environments and illustrates the relevance of the 4 S dimensions within this context.

### Strengths and limitations

This study has several strengths. The use of Schlossberg’s Transition Theory provided a theoretically informed framework for examining educators’ experiences of transition while the combined deductive–inductive analytic approach allowed context-specific aspects of digital HPE to emerge from participants’ accounts. The inclusion of participants from two programme cohorts with diverse professional backgrounds and career stages contributed to a breadth of perspectives on the transition to digital teaching. In addition, the interdisciplinary composition of the research team, iterative analytic discussions, researcher triangulation, and reflexive practices strengthened the rigour and credibility of the analysis.

One limitation of this study concerns its single institution setting, which may affect the generalisability of the findings to other academic and cultural contexts with different faculty development structures, resources, and digital maturity levels. Furthermore, some members of the research team were involved in the conceptualisation and delivery of the DHPE programme, which, despite deliberate reflexive strategies and researcher triangulation, could have influenced data interpretation or participant responses due to potential social desirability bias. Language was also a limitation, as all the interviews and focus groups were conducted and analysed in German, but the quotations were translated into English for publication. Despite careful translation, some nuances of meaning or linguistic style may have been lost, thus limiting the potential for detailed discourse analysis. While this study captures educators’ experiences with respect to transition, it does not provide an in-depth analysis of specific digital tools or technological implementations. These areas may be explored in future research. Future research should also adopt longitudinal designs to follow up with participants beyond the conclusions of faculty development programmes in the field of digital education, for the purpose of exploring how engagement in communities of practice supports the ongoing transition to digital health professions education, and fostering collaboration, knowledge sharing and innovation over time. Multi-institutional and cross-cultural investigations are also needed to understand the influence of contextual factors, such as institutional culture, leadership support and resource availability, on educators’ ability to sustain digital transformation. Finally, mixed methods or longitudinal qualitative approaches would more comprehensively capture transitions in self-efficacy, digital identity and pedagogical practice.

## Conclusions

The transition to the role of a digital health professions educator is multifaceted, as it extends beyond acquiring technical skills to include role transformation and adaptation within institutional and social contexts. Guided by Schlossberg’s transition theory, this study demonstrates how situational factors, personal resources, support networks, and adaptive strategies interact to shape the experiences of educators. Accordingly, faculty development programmes should combine skill-building with mentorship, reflection, and sustained communities of practice to facilitate such transitions, and institutions should provide recognition, resources, and supportive structures to enable lasting digital transformation in HPE.

## Supplementary Information

Supplementary Material 1.

## Data Availability

The dataset collected and analysed during the current study is available from the corresponding author upon reasonable request.
