# Supplementary material for: Navigating the transition to digital health professions education: a qualitative study of faculty development through the lens of Schlossberg’s transition theory
Source: BMC Med Educ. 2026 Nov 13;26:1858. doi: 10.1186/s12909-026-10761-2 (PMC13761080; doi:10.1186/s12909-026-10761-2)
Supplement: Supplementary file 1 [file 12909_2026_10761_MOESM1_ESM.docx]

**Navigating the Transition to Digital Health Professions Education: A Qualitative Study of Faculty Development through the Lens of Schlossberg’s Transition Theory**

**Guide for Focus Group Discussions / Interviews**

I. **Introduction to the Topic / Icebreaker**

- Brief review of the DHPE program to recall its content (module topics, activities, interactions, etc.)
- What motivated you to participate in this program?

### II. **Personal Learning Experience**

- Which specific competencies or skills did you wish to acquire or improve through the program, and how have your abilities developed in this regard?
- Which aspects of the program design (e.g., teaching methods, materials, interactions) helped you most or were particularly effective?
- Were there any challenges or obstacles during your participation? How did you deal with them?
- How did you experience the program overall? How did it affect your teaching practice and your understanding of digital teaching?
- What influence did participation in the program have on your professional identity as an educator?

### III. **Perceived Competence Growth**

- Which competencies or skills did you acquire or improve through participation in the DHPE program?
- Can you name concrete examples or situations where you applied these new competencies?
- What were your main goals when you decided to participate in the program, and were these goals achieved?

### IV. **Transfer of Content into Practice**

- How successfully were you able to apply the knowledge and skills acquired in the program in your teaching practice?
- Were there challenges or obstacles in implementation, and how did you handle them?
- Is there anything you feel is missing for the practical application of what you learned? If so, what?

**V. Program Satisfaction and Framework Conditions**

- How did you like the framework conditions and design of the qualification program?
- Which aspects of the program helped you the most or were particularly effective?
- Which specific content or topics of the qualification program most influenced or inspired you?

**VII. Recommendations and Suggestions for Improvement**

- Which aspects of the program design (e.g., teaching methods, materials, interactions) did you find most helpful or effective?
- What recommendations or advice would you give to future participants of the DHPE program?
- Are there aspects of the program that, in your opinion, could be improved?
- How do you perceive the topic of “digital teaching” at Charité? What works well? Where do you see problems?

**VIII. Summary and Conclusion**

- Summary of key findings
- Are there any further comments or impressions you would like to share?
- Thanks and farewell

**Possible Follow-up and In-depth Questions**

- Could you please elaborate on that?
- Can you give an example of this situation?
- Are there specific experiences or stories that illustrate your answers?
